# Delphi consensus recommendations for preventing and treating cardiac implantable electronic device infections beyond current guidelines

**DOI:** 10.1038/s41598-026-49515-x

**Published:** 2026-04-22

**Authors:** Benito Baldauf, Kerstin Bode, Mauro Biffi, Antonio Rapacciuolo, Giulio Zucchelli, Maria Grazia Bongiorni, Ernesto Casorelli, Gianfranco Mitacchione, Felix Hohendanner, Emanuele Durante Mangoni, Veronica Dusi, Paul William Xavier Foley, Angelo Pan, Giuseppe Arena, Archana Rao, Sebastian Spencker, Alexander Steger, Carlo Tascini, Valerio Zacà, Federico Migliore, Ojan Assadian, Roberto Cemin, Marzia Giaccardi, Hendrik Bonnemeier

**Affiliations:** 1 Christian-Albrechts-University, Kiel, Germany; 2 University of Applied Sciences, Bremerhaven, Germany; 3 Heart Center, Leipzig, Germany; 4 Policlinic S. Orsola-Malpighi, University of Bologna, Bologna, Italy; 5 Federico II University of Naples, Naples, Italy; 6 Ospedale Evangelico Betania, Naples, Italy; 7 Azienda Ospedaliero-Universitaria Pisana, Pisa, Italy; 8 Santa Maria della Misericordia Hospital, Montepulciano, Italy; 9 Spedali Civili di Brescia, Brescia, Italy; 10 German Heart Center Berlin, Berlin, Germany; 11 University Hospital Federico II, Naples, Italy; 12 University Hospital of Turin, Turin, Italy; 13 Great Western Hospitals, Swindon, UK; 14 Azienda Socio Sanitaria Territoriale di Cremona, Cremona, Italy; 15 Nuovo Ospedale delle Apuane, Massa Carrara, Italy; 16 Liverpool Heart and Chest Hospital, Liverpool, UK; 17 Jewish Hospital, Berlin, Germany; 18 University Hospital, Technical University of Munich, Munich, Germany; 19 University Hospital, Udine, Italy; 20 University of Siena, Siena, Italy; 21 University of Padova, Padova, Italy; 22 Azienda Ospedaliera, Università di Padova, Padova, Italy; 23 University of Huddersfield, Huddersfield, UK; 24 University Hospital, Wiener Neustadt, Austria; 25 San Maurizio Regional Hospital, Bolzano, Italy; 26 Ospedale Santa Maria Annunziata, Florence, Italy; 27 University Hospital Meyer, Florence, Italy; 28 Asklepios Hospital Schildautal, Seesen, Germany; 29 University of Applied Sciences Bremerhaven, An der Karlstadt 8, 27568 Bremerhaven, Germany; 30 Medical Faculty, Christian Albrechts University Kiel, 24211 Bremerhaven, Germany

**Keywords:** Cardiac implantable electronic device, Infection, Treatment, Knowledge gaps, Guidelines, Consensus, Expert panel, Recommendations, Cardiology, Diseases, Health care, Medical research

## Abstract

Infections associated with cardiac implantable electronic devices (CIEDs) pose significant clinical challenges due to their life-threatening nature and complex management. Recent advancements in prevention, diagnosis, and treatment have been driven by landmark trials such as PADIT and WRAP-IT, alongside evolving diagnostic tools like [18 F]FDG PET/CT and updated diagnostic criteria (e.g., 2023 Duke-ISCVID). A combination of well-established approaches—such as leadless pacemakers, subcutaneous ICDs, and an antibiotic-eluting envelope—and evolving adjunctive strategies, including a taurolidine-containing antimicrobial agent, incision drapes, fascial plane blocks or double gloving, have broadened the spectrum of options to reduce and potentially mitigate the risk of CIED infection. While devices like leadless pacemakers, subcutaneous ICDs, and antibiotic envelopes are supported by randomized controlled trials, adjunctive measures such as taurolidine rely predominantly on observational data. To address this variation in the strength of available evidence, a modified Delphi consensus process was conducted, bringing together cardiologists and infectious disease experts to define experience-based best practices in areas where current guidelines offer limited or no specific recommendations. The consensus achieved strong agreement (≥ 80%) on key strategies, including mandatory double-gloving to reduce contamination, the use of validated risk models (e.g., PADIT, BLISTER) for tailored infection prevention employing an antibiotic eluting envelope, multidisciplinary decision-making for non-extractable infections and the use of taurolidine solutions as adjuncts to prevent CIED infection. Moderate consensus (60–79%) supported non-delayed reimplantation after lead-related endocarditis and single-session reimplantation in pacing-dependent patients under specific circumstances. However, no consensus was reached on mandating a minimum annual procedural volume (≥ 500 cases) for CIED centers, reflecting concerns about access and operator expertise. The findings emphasize the importance of personalized risk stratification, procedural innovations, and multidisciplinary collaboration in optimizing CIED infection outcomes. Areas requiring further research include the efficacy of iodophor-impregnated drapes, fascial plane blocks, and taurolidine solutions. This consensus provides a pragmatic framework for clinicians, highlighting evidence-informed strategies to mitigate infection risks and improve care for patients undergoing CIED procedures.

**Supplementary Information:**

The online version contains supplementary material available at 10.1038/s41598-026-49515-x.

## Introduction

Infections associated with cardiac implantable electronic devices (CIEDs) represent a significant clinical challenge due to their life-threatening nature and the complexities involved in both diagnosis and management. Among these, lead-related infective endocarditis (L-IE) is a particularly problematic complication, often complicating clinical decision-making and patient outcomes. Since the initial scientific statements issued by key professional bodies such as ECAS, EHRA, AHA, HRS, and CTS, substantial advancements have been made in the prevention, diagnosis, and treatment of CIED infections.^1–6^.

Notably, landmark randomized controlled trials like PADIT^7^ and WRAP-IT^8^ have provided pivotal evidence supporting infection prevention strategies, including the use of antibiotic-impregnated envelopes during device implantation. Diagnostic modalities have also evolved, with critical reassessment of transesophageal echocardiography (TEE) revealing limitations in differentiating infectious from noninfectious lead masses, and the growing utilization of fluorine-18-fludeoxyglucose positron emission tomography/computed tomography ([18 F]FDG PET/CT) enhancing diagnostic accuracy.^2^ The recent update to the 2023 Duke-International Society for Cardiovascular Infectious Diseases criteria further recognizes CIED presence as a specific minor criterion for L-IE diagnosis, reflecting its established role as a predisposing factor.^4^.

In parallel, novel device technologies and adjunctive therapies, including Taurolidine-containing antimicrobial agents^9,10^ leadless pacemakers (LPMs),^5^ extravascular implantable cardioverter defibrillators (EV-ICDs)^11^ and subcutaneous implantable cardioverter defibrillators (S-ICDs)^12^, have expanded therapeutic options. Infection risk scores have gained traction as tools to stratify patients and tailor preventive measures, guiding both device selection and the application of infection mitigation strategies such as antibiotic eluting envelopes^13^. Additionally, emerging techniques like percutaneous mechanical aspiration systems offer minimally invasive approaches to managing complicated right-sided vegetations in select cases^14^.

Building on these advances, this Delphi consensus statement^15^ was developed by an expert panel committed to improving outcomes for patients undergoing CIED procedures through the promotion of evidence-informed best practices aimed at minimizing infection risk. While this statement does not constitute a comprehensive review of all facets of CIED infection, including detailed clinical features, epidemiology, and pathogenesis, nor does it serve as formal clinical guidelines, it provides pragmatic strategies and algorithms designed to support clinicians worldwide in optimizing care for this vulnerable patient population.

## Methods

To develop a consensus statement on the management and prevention of CIED infections, a modified Delphi method was conducted in accordance with the “Reporting guidelines for Delphi techniques in health sciences”.^16^. The expert panel comprised faculty from the 360° CIED Infection Congress, including cardiologists, cardiothoracic surgeons and infectious disease specialists practicing in Italy, Germany, Austria, and the United Kingdom, representing tertiary referral centers with extensive clinical experience and published expertise in device-related infections, who gave consent deliberately to participate in the survey before the survey and during the congress itself.

Invited experts participated in iterative survey rounds conducted anonymously via SurveyMonkey^®^ (SVMK, San Mateo, CA, USA), aiming to achieve consensus on the most critical topics in CIED infection prevention and management.

Several measures were implemented to reduce bias. Participation was voluntary but restricted to clinicians with established expertise in CIED infections. All voting rounds were conducted anonymously, preventing influence from dominant personalities or hierarchical relationships within the panel. Aggregated results were shared between rounds to allow participants to reconsider their responses while maintaining anonymity.

The modified Delphi process consisted of three sequential stages. First, during the 360° CIED Infection Congress, panel members participated in an exploratory round in which key clinical areas lacking clear guideline recommendations were identified and discussed. Based on this discussion, the coordinating group formulated ten preliminary statements reflecting the topics considered most relevant to CIED infection prevention and management.

In the second stage (first formal voting round), panelists rated each statement using a 5-point Likert scale (1 = strongly agree, 5 = strongly disagree). Following this round, aggregated voting results were circulated to participants. The strength of agreement was classified as strong agreement (≥ 80%), moderate agreement (> 55%) or no agreement (< 50%) as extrapolated from the RAND methodology. The results were presented via electronic mail to all participants.

In the third stage (final consensus round), panelists were invited to reconsider the statements in light of the group responses from the first formal voting round. Statements were not substantially modified between rounds, but clarifications were provided where wording had led to uncertainty. The results from this final round were used for the consensus analysis presented in this manuscript. In addition to reporting overall agreement rates (scores 1–2), the distribution of responses across the full 5-point Likert scale was recorded to enhance transparency of voting patterns (Table S1).

Online consensus rounds were conducted anonymously. The results were exported from SurveyMonkey^®^ and analyzed using Microsoft Excel (Microsoft Corporation, Redmond, USA).

In accordance with EU GDPR Recital 26 and the guidance of the Italian “Garante per la Protezione dei Dati Personali”, the data collected qualify as fully anonymized and therefore fall outside the scope of data protection legislation granting a waiver of formal ethical approval.

## Results

Of the 21 members of the 360° CIED Infection Faculty, 20 participated in the final round of the modified Delphi consensus process. Consensus was achieved in nine out of ten questions. The questions and corresponding results are summarized in Table 1. The full distribution of Likert responses for each statement is presented in Table S1 in the Supplementary to provide greater transparency regarding the degree of agreement and dissent among panel members.

**Table 1:** Level of Consensus Among Experts on Clinical Practice Questions Related to CIED Infection Prevention and Management: Responses were categorized as strong consensus (≥ 80% agreement), moderate consensus (55–79% agreement), or no consensus (< 50% agreement). Percentages reflect the proportion of participants who agreed or strongly agreed with each statement.

Question	Strong Consensus (≥ 80)	Moderate (55–79%)	No Consensus (< 50%)
Q1: Double-gloving and changing the outer pair of gloves after draping the patient and before device handling (i.e., new generator, new leads) should be mandatory to reduce contamination risk	81.25% agreed or strongly agreed		
Q2: Established risk models (e.g., PADIT score, BLISTER Score, SHARIFF) are clinically valuable for assessing CIED infection risk and should be applied routinely across all clinical settings to guide the appropriate use of antibiotic-eluting envelopes during device implantation.	88.23% agreed or strongly agreed		
Q3 In case of CIED infection in a frail patient, a patient not willing to undergo hardware extraction or in case lead extraction is impossible or unfeasible, the option of revision including antimicrobial irrigation and thorough debridement, with generator replacement in subpectoral position (Giudice et al., Weichsel et al., Borov et al., Giaccardi et al., Casorelli et al.) or through continuous ultra-high in situ targeted antibiotic therapy in the absence of debridement (Topaz et al.) should be discussed within the heart team, the patient and the patient’s relatives. (after securing the diagnosis of localised CIED pocket infection ONLY and in extracting centers SOLELY! ).	88.23% agreed or strongly agreed.		
Q4 Reimplantation of a CIED after extraction due to a lead-related endocarditis is feasible even after one week if blood-cultures remain negative.		70.59% agreed or strongly agreed	
Q5 Single session reimplantation of a CIED after extraction due to localised CIED infection (blood culture and TEE study negative) in contralateral position is feasible in pacing dependent individuals.		70.59% agreed or strongly agreed. 17.65% neutral rate	
Q6 CIED implantation should be limited to centers with an annual procedural volume of at least 500 cases to ensure optimal outcomes and procedural safety.			Disagreement (47.05%) outweighs agreement (41.18%)
Q7 Fascial plane blocks (how to) should be routinely employed during CIED implantation to improve postoperative comfort and reduce the risk of patient interference with the surgical site.		58.82% agreed or strongly agreed; 29.41% neutral rate	
Q8 The use of iodophor-impregnated incision drapes should be standard practice during CIED implantation procedures to reduce microbial skin flora and lower the risk of surgical site infection.		58.82% agreed or strongly agreed; 35.29% neutral	
Q9 Taurolidine-containing solutions are certified for adjunct use during CIED procedures, they have proven to be safe (Vonthein et al., Borov et al.) and may be considered as adjuncts in infection prevention for any CIED procedure.	88.24% agreed or strongly agreed		
Q10 Strap fixation should be routinely used to stabilize the patient on the procedure table during CIED implantation in order to minimize involuntary movement, reduce the risk of lead dislodgement, and prevent contamination of the surgical field.		64.71% agreed or strongly agreed. 17.65% neutral rate	

## Discussion

### Double-gloving protocol (Q1)

A strong consensus (81.25% agreement) supports mandatory double-gloving with glove changes post-draping to minimize contamination risk. Double-gloving—wearing two pairs of sterile surgical gloves—has been extensively studied for its role in reducing glove perforation rates and enhancing infection control during surgical procedures. A comprehensive Cochrane review analyzed 14 trials and found that double-gloving significantly reduces perforations to the innermost gloves, with an odds ratio of 4.10 (95% CI 3.30 to 5.09) compared to single gloves.^17^ This reduction in glove breaches is crucial for minimizing the risk of pathogen transmission between the surgical team and the patient. Further supporting these findings, a systematic review encompassing 18 randomized controlled trials reported that double-gloving decreases blood contamination risk by 65% and inner glove perforation by 71% compared to single gloves.^18^ Additionally, the use of indicator gloves—colored latex gloves worn underneath standard gloves—has been shown to significantly improve the detection of perforations during surgery, enhancing the ability to maintain sterility.^19^ Despite these advantages, the adoption of double-gloving is not universal in all surgical disciplines, including CIED procedures. Concerns regarding manual dexterity and increased resource utilization are valid; however, studies suggest that double-gloving does not significantly impair surgical performance. Implementing double-gloving protocols may require adjustments in training and workflow to ensure that the benefits outweigh the costs.

### Use of risk models (Q2)

With 88.23% agreement, there is strong endorsement for employing validated infection risk prediction models such as PADIT,^7^ BLISTER,^13^ or SHARIFF^20^ to guide antibiotic envelope use and tailor prophylaxis. These models reflect an evolving shift toward personalized medicine in CIED infection prevention, enabling stratification of patients by risk and more judicious antibiotic use, which is critical to mitigate antimicrobial resistance. Current studies support these tools’ predictive accuracy, yet their clinical utility depends on ease of integration into routine practice and ongoing validation across diverse patient cohorts.^21^.

### Alternative treatment pathways in non-extractable infections (Q3)

The consensus (88.23% agreement) favors heart team–led multidisciplinary decision-making incorporating patient and family preferences for managing complex CIED infections where device extraction is not feasible. Alternative approaches like targeted ultra-high in-situ antibiotic administration^22^ or capsulectomy, thorough debridement and taurolidine-containing solution antisepsis with subpectoral repositioning^23–27^ align with emerging clinical practice emphasizing individualized care plans, especially in frail or anatomically challenging patients. This paradigm prioritizes balancing infection control with quality of life and procedural risk, as supported by contemporary case series and observational data. The panel noted that this statement encompassed several complex clinical scenarios (frailty, non-extractable systems, and alternative infection management strategies). While agreement remained strong, the breadth of the statement may limit interpretability and highlights the need for future work to address these scenarios individually.

### Reimplantation after lead-related endocarditis (Q4)

A reasonable consensus (70.59% agreement) supports device reimplantation as early as one week following blood culture clearance in lead-related endocarditis cases. This aligns with current infectious disease guidelines that recommend ensuring infection resolution prior to reimplantation to reduce relapse risk, but lack clear guidance on timing.^4,5^ The minority expressing neutral or dissenting views reflect variability in institutional protocols or risk tolerance, underscoring the need for additional prospective studies to optimize courageous timing supported here.

### Single-session reimplantation after local infection (Q5)

Support for contralateral single-session reimplantation in pacing-dependent patients (70.59% agreement) reflects clinical pragmatism when strict sterile conditions and complete microbiological workup are ensured. However, a notable neutral rate (17.65%) suggests ongoing debate regarding the balance between infection control and urgent pacing needs. Given the limited existing evidence, it’s crucial to prioritize individualized assessments including proactive prevention strategies, and adherence to rigorous infection screening.

### Minimum center volume requirement (Q6)

 Disagreement predominates (47.05% vs. 41.18% agreement) regarding a minimum annual procedural volume (≥ 500 CIED cases) for centers performing procedures such as CIED implants, revisions and extractions. The lack of consensus reflects complex factors: operator skill and team experience may outweigh absolute procedural numbers, and arbitrary cutoffs risk limiting access to care. Literature indicates volume–outcome relationships but fails to define precise thresholds, highlighting the need for nuanced quality metrics beyond volume alone.^5^.

### Routine use of fascial plane blocks (Q7)

Moderate support (58.82% agreement, 29.41% neutral) exists for fascial nerve blocks as adjuncts to improve pain control and potentially reduce infection risk through decreased wound manipulation.^28^ While promising data from analogous surgical disciplines exist, specific evidence in CIED populations remains limited. Training requirements and procedural logistics further temper enthusiasm, pointing to the need for focused clinical trials to define safety, efficacy, and standardized protocols.

### Iodophor-impregnated drapes (Q8)

Moderate support (58.82% agreement, 35.29% neutral) was noted for iodophor-impregnated drapes. These antimicrobial drapes have demonstrated efficacy in reducing surgical site infections across multiple specialties, attributed to sustained local antiseptic action. However, neutrality among panelists likely reflects concerns about increased costs, resource implications, and inconsistent evidence specific to CIED procedures when employed adjunctively to standard sterile draping. While cost-effectiveness analyses are sparse, available data suggest potential benefit, warranting further large-scale comparative trials to clarify their role and facilitate cost-benefit assessments in this specific setting.^29^.

### Use of taurolidine solutions (Q9)

Strong consensus (88.24%) endorses taurolidine-containing antimicrobial agents as valuable adjuncts in infection prevention. Taurolidine is a synthetic taurine derivative with broad antimicrobial activity against gram-positive, gram-negative, and fungal organisms, as well as anti-biofilm properties. It has been widely used in Europe in the prevention of catheter-related infections and has more recently been investigated as an adjunctive antiseptic strategy during CIED procedures^9,10^ Despite promising results, the panel appropriately calls for more data to confirm definitive clinical efficacy before recommendation in all CIED procedures.

### Patient fixation during implantation (Q10)

Although seemingly procedural in nature, patient stabilization during device implantation was included because unintended patient movement may compromise sterile conditions and procedural safety, particularly in procedures performed under conscious sedation. Reasonable support (64.71% agreement) exists for routine use of patient strap fixation to reduce patient movement during CIED procedures ideally minimizing lead dislodgement and contamination risk. While patient fixation during CIED procedures is widely regarded as a best practice, some variability in neutral responses suggests differences in procedural habits or patient-specific considerations. Current evidence from observational studies supports strap fixation as a simple, effective measure to enhance procedural outcomes and should be further evaluated in controlled studies.

## Limitations

Several limitations inherent to the Delphi methodology should be acknowledged. First, although the panel included cardiologists, cardiac surgeons, and infectious disease specialists with recognized expertise in CIED infections, the expert group was relatively small and consisted primarily of European clinicians, which may limit the generalizability of the findings to other healthcare systems and practice environments.

Second, Delphi consensus reflects expert opinion rather than direct clinical evidence and may therefore be influenced by prevailing clinical experience and local practice patterns. While anonymity of voting was maintained to reduce peer influence and dominance bias, residual bias cannot be fully excluded.

Third, the statements were generated by the expert panel itself, which may introduce topic selection bias toward areas perceived as clinically relevant by the panel rather than representing all unresolved issues in CIED infection management.

Fourth, the consensus process does not replace guideline development based on systematic evidence review, but instead provides experience-based guidance in areas where robust clinical trial data remain limited.

Finally: Practice patterns, resource availability, and regulatory environments vary substantially between regions; therefore, some recommendations discussed here may not be directly transferable to all healthcare systems.

## Conclusion

This consensus statement reflects the collective opinion of a multidisciplinary panel of experts regarding practical approaches to CIED infection prevention and management in areas where guideline recommendations remain limited. Through a systematic exploration of critical topics, including procedural best practices, patient-specific risk stratification, emerging prophylactic adjuncts, and institutional quality benchmarks, this consensus seeks to advance clinical decision-making with strategies that are both evidence-informed and pragmatically applicable. Importantly, areas of only moderate agreement, such as the use of iodophor-impregnated drapes and fascial plane blocks, reflect ongoing uncertainties and underscore the need for targeted research to refine and validate these practices. This evolving body of knowledge highlights the dynamic nature of CIED infection prevention and the necessity of continual reassessment as new data emerge.

## Supplementary Information

Below is the link to the electronic supplementary material.

Supplementary Material 1

## Data Availability

All relevant data generated or analyzed during this study are included within this manuscript. No additional datasets were generated or used.
