# Supplementary material for: Delphi consensus recommendations for preventing and treating cardiac implantable electronic device infections beyond current guidelines
Source: Sci Rep. 2026 Apr 22;16:13135. doi: 10.1038/s41598-026-49515-x (PMC13102974; doi:10.1038/s41598-026-49515-x)
Supplement: Supplementary file 1 — Supplementary Material 1 [file 41598_2026_49515_MOESM1_ESM.docx]

| Table S1: Percentages of agreement and disagreement obtained during the Delphi consensus process. | | | | | |
| --- | --- | --- | --- | --- | --- |
| Question | Strongly agree | Agree | Neutral | Disgagree | Strongly disagree |
| Q1: Double-gloving and changing the outer pair of gloves after draping the patient and before device handling (i.e., new generator, new leads) should be mandatory to reduce contamination risk | 56.25 | 25.0 | 6.25 | 12.5 | 0.0 |
| Q2: Established risk models (e.g., PADIT score, BLISTER Score, SHARIFF) are clinically valuable for assessing CIED infection risk and should be applied routinely across all clinical settings to guide the appropriate use of antibiotic-eluting envelopes during device implantation. | 35.29 | 52.94 | 5.88 | 5.88 | 0.0 |
| Q3 In case of CIED infection in a frail patient, a patient not willing to undergo hardware extraction or in case lead extraction is impossible or unfeasible, the option of revision including antimicrobial irrigation and thorough debridement, with generator replacement in subpectoral position (Giudice et al., Weichsel et al., Borov et al., Giaccardi et al., Casorelli et al.) or through continuous ultra-high in situ targeted antibiotic therapy in the absence of debridement (Topaz et al.) should be discussed within the heart team, the patient and the patient's relatives. (after securing the diagnosis of localised CIED pocket infection ONLY and in extracting centers SOLELY!). | 29.41 | 58,82 | 5.88 | 0.0 | 5.88 |
| Q4 Reimplantation of a CIED after extraction due to a lead-related endocarditis is feasible even after one week if blood-cultures remain negative. | 17.65 | 52.94 | 11.76 | 11.76 | 5.88 |
| Q5 Single session reimplantation of a CIED after extraction due to localised CIED infection (blood culture and TEE study negative) in contralateral position is feasible in pacing dependent individuals. | 23.52 | 47.06 | 17.65 | 11.76 | 0.0 |
| Q6 CIED implantation should be limited to centers with an annual procedural volume of at least 500 cases to ensure optimal outcomes and procedural safety. | 17.65 | 23.53 | 11.76 | 35.29 | 11.76 |
| Q7 Fascial plane blocks (how to) should be routinely employed during CIED implantation to improve postoperative comfort and reduce the risk of patient interference with the surgical site. | 5.88 | 52.94 | 29.41 | 5.88 | 5.88 |
| Q8 The use of iodophor-impregnated incision drapes should be standard practice during CIED implantation procedures to reduce microbial skin flora and lower the risk of surgical site infection. | 23.53 | 35.29 | 35.29 | 5.88 | 0.0 |
| Q9 Taurolidine-containing solutions are certified for adjunct use during CIED procedures, they have proven to be safe (Vonthein et al., Borov et al.) and may be considered as adjuncts in infection prevention for any CIED procedure. | 41.18 | 47.06 | 5.88 | 5.88 | 0.0 |
| Q10 Strap fixation should be routinely used to stabilize the patient on the procedure table during CIED implantation in order to minimize involuntary movement, reduce the risk of lead dislodgement, and prevent contamination of the surgical field. | 23.53 | 41.18 | 17.65 | 11.76 | 5.88 |
